# The association between serum 25-hydroxyvitamin D levels and retinopathy of prematurity in preterm infants

**DOI:** 10.3389/fped.2024.1404196

**Published:** 2024-08-02

**Authors:** Xiangyun Yin, Shimin Xu, Xuefei Zhang, Liangliang Li, Hongmin Xi, Lili Ma, Mengya Sun, Ping Yang, Xianghong Li, Hong Jiang

**Affiliations:** ^1^ Department of Neonatology, The Affiliated Hospital of Qingdao University, Qingdao, China; ^2^ Department of Neonatology, Beijing Jingdu Children’s Hospital, Beijing, China; ^3^ Qingdao University, Qingdao, China

**Keywords:** retinopathy of prematurity, serum 25-hydroxyvitamin D, lower birth weight infants, preterm infant, sepsis

## Abstract

**Objective:**

This study aimed to investigate the correlation between serum 25-hydroxyvitamin D (25(OH)D) levels and retinopathy of prematurity (ROP) in premature infants one month after birth.

**Methods:**

Preterm infants (gestational age <32 weeks) admitted to the Affiliated Hospital of Qingdao University from 2017 to 2022 were divided into ROP and non-ROP groups based on ROP occurrence any stage. Serum 25(OH)D levels and clinical data were compared between the two groups at 1 month after birth, and the relationship between vitamin D levels and ROP was analyzed.

**Results:**

Among the 217 premature infants included, 55 (25.35%) were in the ROP group, and 162 (74.65%) were in the non-ROP group. The ROP group had lower gestational age and birth weight, longer invasive ventilation (IV), non-invasive ventilation (NIV), and oxygen therapy times compared to the non-ROP group. Apgar scores, cesarean delivery, and antenatal steroids ratios were lower in the ROP group, while sepsis and pulmonary surfactant utilization ratios were higher (all *p* < 0.05). Significant differences in serum 25-(OH)D levels were observed among children in the non-ROP group (14.20 ± 5.07 ng/ml), ROP treated group (7.891 ± 1.878 ng/ml), and untreated group (12.168 ± 4.354 ng/ml) (*p* < 0.001). Multivariate regression analysis identified antenatal steroids as protective factors and lower birth weight, serum 25-(OH)D levels, long-term invasive mechanical ventilation, and sepsis as independent risk factors for ROP in premature infants.

**Conclusion:**

Vitamin D, lower birth weight, long-term invasive mechanical ventilation, and sepsis were associated with incidence of ROP in preterm infants. Vitamin D was associated with the severity of ROP, emphasizing the importance of prudent vitamin D supplementation and regular monitoring of serum 25-(OH)D levels.

## 1 Introduction

Retinopathy of prematurity (ROP) is characterized by abnormal proliferation of retinal blood vessels in the eyes of premature infants. The incidence and severity of ROP increase with decreasing gestational age and birth weight, posing a significant threat to infant vision. Severe cases may lead to retinal detachment and subsequent blindness (1–4). ROP is linked to elevated levels of vascular endothelial growth factor (VEGF) caused by local hypoxia, promoting aberrant formation of blood vessels in newborns. After birth, premature infants experience a deficiency in the supply of IGF-1 from the placenta and lack autonomous production, thereby impacting the development of retinal blood vessels. Key factors contributing to ROP include premature birth, low birth weight, oxygen therapy, anemia, blood transfusions, sepsis, metabolic acidosis, respiratory distress syndrome (RDS), apnea, and maternal pregnancy complications (5, 6). As our understanding of ultra-premature infants advances, early identification of high-risk factors for ROP becomes crucial for prevention and treatment, ultimately reducing the incidence of blindness and improving the quality of life for premature infants (7).

Vitamin D, a biologically active fat-soluble vitamin synthesized endogenously, not only regulates calcium and phosphorus metabolism but also exhibits antioxidant and anti-inflammatory effects. Additionally, it plays a role in anti-angiogenesis, cell growth, and differentiation. Eye tissues contain vitamin D receptors and 1-hydroxylase, indicating a local regulatory role (8). Given that placental transfer of vitamin D mainly occurs during the third trimester, vitamin D deficiency is a concern, particularly in premature infants with a gestational age <32 weeks (9, 10). However, there is limited research on the association between vitamin D and ROP. Some studies suggested that lower 25(OH)D levels in premature infants with a gestational age <32 weeks after birth might be related to ROP (11–13).

This study aims to collect 25(OH)D levels in premature infants with a gestational age <32 weeks at 1 month after birth and retrospectively analyze clinical data to explore the relationship between serum 25(OH)D levels and ROP. The findings provide valuable insights for the clinical prevention and treatment of ROP in premature infants.

## 2 Patients and methods

### 2.1 Preterm infants

A retrospective study was conducted on all preterm infants with a gestational age <32 weeks admitted to the intensive care unit at the Affiliated Hospital of Qingdao University during 2017–2022. All infants in the hospital signed the written consent “clinical samples to carry out scientific research” by parents.

Inclusion criteria: (1) Preterm infants with a gestational age <32 weeks. (2) Premature infants admitted to the hospital within 24 h after birth, with a hospitalization duration exceeding 4 weeks. (3) Infants whose condition improves or is cured upon discharge.

Exclusion criteria: (1) Specific congenital eye diseases, such as retinoblastoma, congenital cataract, glaucoma, etc. (2) Infants with genetic metabolic diseases and congenital developmental malformations. (3) Cases involving death during hospitalization, incomplete clinical data, or family members opting for treatment discontinuation. (4) Individuals with incomplete information.

Based on the occurrence of retinopathy of prematurity (ROP), participants were divided into the ROP group and non-ROP group. Within the ROP group, two subgroups were identified based on whether ranibizumab or laser treatment was administered: treated ROP group and untreated group. The study received approval from the Affiliated hospital of Qingdao University ethics committee (QYFY WZLL 28884).

### 2.2 Methods

#### 2.2.1 Vitamin D supplementation method

Enteral feeding should be initiated once the condition of the included children stabilizes. Refer to the “Recommendations for the Prevention and Treatment of Vitamin D Deficiency and Vitamin D Deficiency Rickets” (14) for vitamin D supplementation guidance. For preterm infants with a gestational age <32 weeks, prioritize breast milk when establishing enteral feeding. Breast milk fortifier(Similac Human Milk Fortifier, POWDER) is added when milk intake reaches 80–100 ml/kg. If breast milk is insufficient, formula milk is provided. Daily supplements include 1 Vitamin AD Soft Capsules (DYNE PHARMA, vitamin D 500 IU) and vitamin D3 400 IU per day. Parenteral nutrition for all premature infants follows the 2013 Chinese Clinical Application Guidelines for Neonatal Nutritional Support (15), with the vitamin D content of fat-soluble vitamins in intravenous nutrition at 400 IU/10 ml (neonatal dosage is 1 ml/kg).

#### 2.2.2 ROP screening

Follow the “China Retinopathy of Prematurity Screening Guidelines (2014)” (16). Qualified ophthalmologists use indirect ophthalmoscopy or RetCam fundus cameras to screen infants with a gestational age of 32 weeks or a birth weight of 2,000 g. Premature and low birth weight infants undergo ROP screening, with the first screening performed at 4–6 weeks after birth or 31–32 weeks after menstruation, until corrected the gestational age to 44 weeks, retinal blood vessels should grow until the jagged edge. Indications for early treatment of ROP (17) involve the use of anti-VEGF drug ranibizumab or laser therapy.

#### 2.2.3 Data collection methods: collect data on infants and their mothers through electronic medical records

General information includes sex, gestational age, birth weight, mode of delivery, Apgar scores at 1 and 5 min.

Clinical data encompass oxygen therapy time, invasive ventilation time, non-invasive ventilation time, serum 25-(OH)D level at 1 month after birth, pulmonary surfactant (PS) treatment and breastfeeding time.

Perinatal factors include preeclampsia, gestational diabetes mellitus, and antenatal steroids.

Major complications are defined as follows: Bronchopulmonary dysplasia (BPD), Patent ductus arteriosus (PDA), necrotizing enterocolitis (NEC), and sepsis.

Use of invasive ventilation, non-invasive ventilation, PS, and antenatal steroids aligns with the “European Consensus Guidelines for the Management of Neonatal Respiratory Distress Syndrome (2019 Edition)” (18).

Oxygen therapy time is calculated by summing mechanical ventilation time, hood oxygen inhalation time, and nasal cannula oxygen inhalation time.

## 3 Statistical methods

SPSS 29.0 statistical software was utilized for comprehensive data analysis, while GraphPad Prism 10 software facilitated graphical representation. For measurement data conforming to a normal distribution, mean ± standard deviation (*x* ± *s*) was employed. Group comparisons were conducted using independent samples *t*-test and single-factor ANOVA. In cases where measurement data deviated from normal distribution, the results are presented as median (interquartile range) M (Q1, Q3), and the Mann–Whitney *U* rank sum test was applied for between-group comparisons.

Count data was expressed as case (percentage) *n* (%), and the *χ*^2^-test or Fisher's exact probability method was employed for group comparisons. Single-factor analysis helped identify statistically significant influencing factors as independent variables, with ROP occurrence serving as the dependent variable in the logistic regression model for multi-factor analysis. A significance threshold of *p* < 0.05 was considered indicative of statistically significant differences.

## 4 Results

### 4.1 Demographic information

Throughout the study period, 217 premature infants with a gestational age <32 weeks met the inclusion criteria at our hospital. This cohort comprised 162 infants (74.65%) in the non-ROP group and 55 infants (25.35%) in the ROP group. Specifically, among the ROP group, 13 infants underwent treatment with ranibizumab (treated ROP group), constituting 23.6% (13/55), while 42 infants remained untreated, representing 76.4% (42/55).

### 4.2 Single factor analysis of ROP

#### 4.2.1 Analysis of perinatal factors

In the ROP group, the gestational age, birth weight, Apgar score, rate of cesarean section, and antenatal steroid usage were all notably lower than those observed in the non-ROP group. These differences reached statistical significance (*p *< 0.05). In contrast, when comparing sex, gestational diabetes mellitus, and preeclampsia between the two groups, no statistically significant differences were identified (*p *> 0.05) (refer to Table 1).

**Table 1:** Comparison of perinatal factors between the two groups.

	non-ROPgroup (162)	ROP group (55)	Statistical value	*p*-value
Sex			0.357	0.55
Female^a^	72 (44.4)	27 (49.1)		
Male^a^	90 (55.6)	28 (50.9)		
GA (d)^b^	212 (204,219)	196 (186,206)	−6.701	<0.001
BW (g)^b^	1,300 (1,100,1,550)	970 (850,1,130)	−6.722	<0.001
Cesarean delivery^a^	132 (81.5)	34 (61.8)	8.83	0.003
Apgar 1 min^b^	8 (7,9)	6 (5,8)	−4.923	<0.001
Apgar 5 min^b^	9 (8,10)	8 (7,9)	−4.661	<0.001
GDM^a^	28 (17.3)	7 (12.7)	0.63	0.427
Preeclampsia^a^	60 (37.0)	13 (23.6)	3.303	0.069
Antenatal steroids^a^	113 (69.8)	19 (34.5)	21.361	<0.001

GA, gestational age; BW, birth weight; GDM, gestational diabetes mellitus.

^a^ Is represented by example (%).

^b^ Is represented by M (Q1, Q3), and the statistical value is *Z* value or *X*^2^ value.

#### 4.2.2 Clinical data analysis

The serum 25-(OH)D level in the ROP group was observed to be lower than that in the non-ROP group. Additionally, the ROP group exhibited significantly higher values in terms of invasive ventilation time, non-invasive ventilation time, oxygen therapy time, sepsis incidence, and pulmonary surfactant (PS) treatment compared to the non-ROP group, with all differences proving statistically significant (*p *< 0.05). In contrast, there were no statistically significant differences noted in the occurrences of Patent Ductus Arteriosus (PDA), Bronchopulmonary Dysplasia (BPD), and Necrotizing Enterocolitis (NEC) between the two groups (*p *> 0.05) (refer to Table 2).

**Table 2:** Comparison of clinical data between the two groups.

	non-ROP group (162)	ROPgroup (55)	Statistical value	*p-*value
25- (OH) D (ng/ml)^a^	14.20 ± 5.07	11.16 ± 4.31	3.982	<0.001
IV time (d)^b^	0 (0,0)	1 (0,11)	−6.634	<0.001
NIV time (d)^b^	11.5 (7,31)	31 (12,53)	−4.017	<0.001
Days with oxygen (d)^b^	7, (19,47.25)	35 (53,79)	−5.818	<0.001
PDA^c^	65 (40.1)	24 (43.6)	0.209	0.647
BPD^c^	53 (32.7)	22 (40.0)	0.963	0.326
Sepsis^c^	15 (9.3)	19 (34.5)	19.869	<0.001
PS^c^	50 (30.9)	34 (61.8)	16.582	<0.001
NEC^c^	17 (10.5)	9 (16.4)	1.341	0.247
DOB (d)^b^	51.5 (15,68.13)	38.5 (9,61)	−1.644	0.1

IV, invasive ventilation; NIV, non-invasive ventilation; PDA, patent ductus arteriosus; BPD, bronchopulmonary dysplasia; PS, pulmonary surfactant; NEC, necrotizing enterocolitis; DOB, days of breastfeeding.

^a^ Is expressed as *x* ± *s*.

^b^ Is expressed as M (Q1, Q3), and the statistical value is *t*, *Z* or X^2^ value.

^c^ Is expressed as example (%).

#### 4.2.3 Multifactor analysis of ROP risk factors

Multivariate logistic regression analysis was employed on variables that exhibited statistical significance in the univariate analysis. The outcomes revealed that antenatal steroids serve as a protective factor for ROP in premature infants. Conversely, lower vitamin D, lower birth weight, prolonged invasive mechanical ventilation time, and sepsis were identified as independent risk factors for the development of retinopathy of prematurity (ROP) in preterm infants (refer to Table 3).

**Table 3:** Logistic regression analysis of risk factors for retinopathy of prematurity.

Influence factor	*B*	Wald value	*p-*value	OR value	95% CI
Level of 25- (OH) D	−0.105	4.471	0.034	0.901	0.818–0.992
BW	−0.003	7.91	0.005	0.997	0.994–0.999
Sepsis	1.102	4.131	0.042	3.011	1.04–8.717
Antenatal steroids	−1.821	14.846	<0.001	0.162	0.064–0.409
IV time	0.109	6.733	0.009	1.115	1.027–1.211

#### 4.2.4 Comparison of serum 25-(Oh)D levels among non-ROP, treated ROP, and untreated ROP groups and correlation analysis with ROP severity

**Figure 1:**
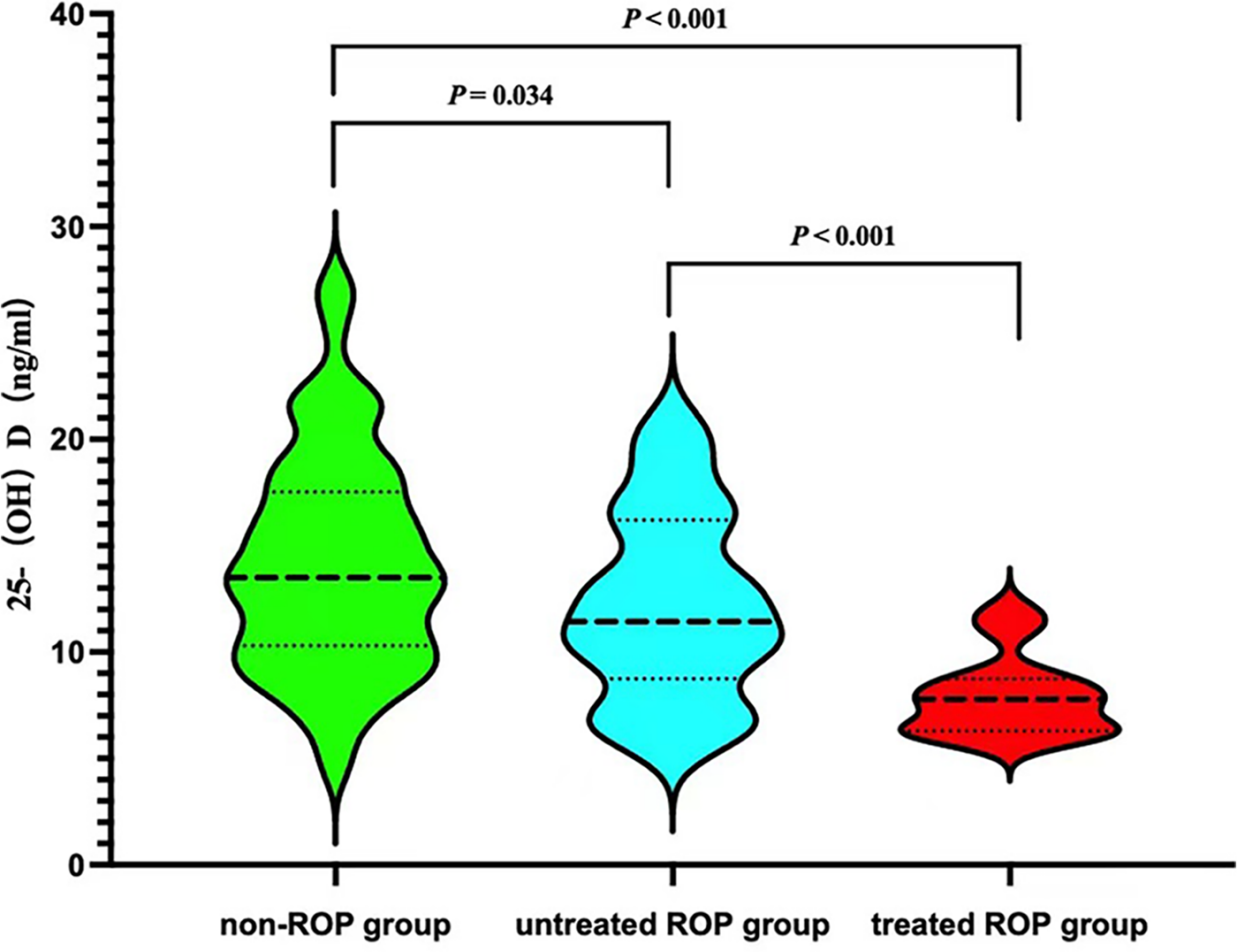
Comparison 25- (OH) D levels among three groups.

Significant differences in serum 25-(OH)D levels were observed among the three groups of children (*p *< 0.001). Specifically, the treated ROP group exhibited the lowest serum 25-(OH)D levels (see Figure 1).

## 5 Discussion

ROP is one of the leading causes of blindness in preterm infants. The development of fetal retinal blood vessels initiates from the optic disc at approximately 16 weeks of gestation and progresses towards the periphery. By about 32 weeks of gestation, these vessels reach the lateral peripheral part. Notably, the temporal retinal blood vessels do not achieve full maturity until the fetus reaches full term. The immaturity of retinal blood vessels is more pronounced in infants with younger gestational ages, correlating with an increased likelihood of ROP development. In this study, the incidence of ROP in premature infants with a gestational age <32 weeks was found to be 25.35%, a figure that aligns closely with the results reported in both domestic and foreign studies (16, 19–21).

The etiology and pathogenesis of Retinopathy of Prematurity (ROP) involve the intricate interplay of multiple factors. Notably, preterm birth and low body weight stand out as foundational contributors to the development of ROP (19, 21). Cytokines that play a crucial role in promoting retinal development are relatively scarce in early pregnancy and do not witness a significant increase until the later stages of pregnancy. Among these cytokines, insulin-like growth factor-1 (IGF-1) emerges as a key regulator of the vascular endothelial system, playing a pivotal role in the occurrence and progression of ROP (22, 23). After birth, premature infants experience a deficiency in the supply of IGF-1 from the placenta and lack autonomous production, thereby impacting the development of retinal blood vessels. The findings of this study corroborate these insights, revealing that children in the ROP group had lower gestational ages and birth weights compared to those in the non-ROP group. It is evident that low birth weight serves as a high-risk factor for the onset of ROP.

Due to the underdeveloped respiratory system and nervous system of premature infants, they often experience frequent apnea or neonatal respiratory distress syndrome, necessitating high-concentration oxygen therapy for survival. The immature retinal blood vessels in these infants are highly sensitive to oxygen, and exposure to high concentrations can lead to vasoconstriction and, in severe cases, occlusion, resulting in relative hypoxia in the retina. The intricate interplay of various active factors under these conditions promotes the generation of numerous new blood vessels, culminating in the development of Retinopathy of Prematurity (ROP) (24, 25). The findings of this study align with these mechanisms, indicating that children with ROP had higher durations of invasive mechanical ventilation, non-invasive mechanical ventilation, and oxygen therapy compared to those without ROP. Notably, invasive ventilation time emerged as an independent risk factor for the occurrence of ROP. de las Rivas Ramírez et al. retrospectively collected ROP data from preterm infants treated in Regional University Hospital of Málaga, in the multivariate analysis, weight and mechanical ventilation duration, and late-onset sepsis were independently associated with the development of ROP (26).

This study identifies sepsis as a significant risk factor for Retinopathy of Prematurity (ROP). Sepsis poses an increased risk for ROP, with inflammatory factors playing a pivotal role in this association (27, 28). During infections, premature infants release substantial amounts of cytokines, including IL-6 and TNF-α. These cytokines can inflict damage on endothelial cells or disrupt vascular function, subsequently stimulating retinal vascular endothelial cells to produce vascular endothelial growth factor (VEGF), thereby contributing to the onset of ROP (29–31). Infections in premature infants may also induce hemodynamic changes, influencing retinal blood perfusion and resulting in heightened retinal ischemia, ultimately leading to the development of ROP.

Antenatal steroids emerge as protective factors against Retinopathy of Prematurity (ROP), effectively reducing the risk of its occurrence (32, 33). Notably, this study revealed a notably low rate of antenatal steroid administration in the ROP group, standing at only 34.5%. The “European Guidelines for the Management of Neonatal Respiratory Distress Syndrome” advocate for the administration of antenatal steroids to all pregnant women with a gestational age of less than 34 weeks and those at risk for preterm birth. The administration of glucocorticoids aids in promoting fetal lung maturation, decreasing the postpartum oxygen requirement, and consequently mitigating the risk of ROP. Additionally, antenatal steroids exhibit inhibitory effects on the production of oxidative stress and inflammatory factors, indirectly contributing to the prevention of ROP.

Vitamin D undergoes hydroxylation in the human liver to form 25-(OH)D. This metabolite, characterized by a stable structure and a long half-life, serves as the optimal indicator for assessing the nutritional status of vitamin D (34). Studies by Alsalem et al. (8) have identified the presence of vitamin D receptors and hydroxylase in ocular cells, including corneal epithelial and retinal cells. These cells have the capacity to convert vitamin D from an inactive form to its active form. Given that intrauterine placental transport of vitamin D predominantly occurs in the third trimester, vitamin D deficiency is prevalent among premature infants. Despite routine vitamin D supplementation, our study reveals an overall deficiency in 25-(OH)D levels among premature infants in our hospital. The serum 25-(OH)D concentration in the ROP group at one month is significantly lower than that in the non-ROP group, exhibiting a consistent pattern, and that had significant difference between treated group and untreated group. A study conducted in Iran (35) found an association between low vitamin D levels in premature infants on the first day after birth and ROP. The severity of ROP increased with greater vitamin D deficiency. Another study from India (36) demonstrated persistent vitamin D deficiency in premature infants with ROP at 4 weeks after birth compared to those without ROP. The current understanding of the pathogenesis of vitamin D in retinopathy encompasses several aspects: Antioxidant effect: Vitamin D's antioxidant properties mitigate oxidative stress damage to the retina and optic nerve by preserving mitochondrial function and neutralizing free radicals (37). Anti-inflammatory and immunomodulatory effects: Studies have demonstrated that vitamin D can modulate T cell subset proportions in retinopathy, inhibit inflammatory response signaling pathways, and decrease inflammatory cell infiltration, thereby reducing damage to the retina and optic nerve (38, 39). Anti-angiogenic effect: Deregulation of vascular endothelial growth factor (VEGF) levels leads to (ROP). VIT-D binding domains are present in the promoter regions of VEGF, thus, VIT-D supplementation might aid in restoring VEGF levels, especially in pathologic conditions secondary to low VEGF levels (40). Jamali et al. (41) have demonstrated that vitamin D's inhibitory effect on retinal neovascularization is dependent on the expression of vitamin D receptors. Experimental studies in a mouse model of oxygen-induced ischemic retinopathy revealed that mice treated with calcitriol exhibited a significant decrease in VEGF expression, retinal neovascularization, and blood-retinal barrier permeability (42, 43). Investigation of serum vitamin D levels is recommended, Early correction of vitamin D deficiency may lead to reduction of RoP in premature infants (35).

## 6 Conclusion

In conclusion, this study highlights that Vitamin D, lower birth weight, long-term invasive mechanical ventilation, and sepsis were associated with incidence of ROP in preterm infants.

Standardizing the administration of antenatal steroids, providing judicious vitamin D supplementation, and closely monitoring serum 25-(OH)D levels can be instrumental in mitigating the incidence and severity of ROP in this vulnerable population.

## Data Availability

The raw data supporting the conclusions of this article will be made available by the authors, without undue reservation.
